# Cardiac procedures in ST-segment-elevation myocardial infarction - the influence of age, geography and Aboriginality

**DOI:** 10.1186/s12872-020-01487-0

**Published:** 2020-05-14

**Authors:** Lee K. Taylor, Michael A. Nelson, Marianne Gale, Judy Trevena, David B. Brieger, Scott Winch, Michelle A. Cretikos, Leah A. Newman, Hai N. Phung, Steven C. Faddy, Paul M. Kelly, Kerry Chant

**Affiliations:** 1 Centre for Epidemiology and Evidence, NSW Ministry of Health, Sydney, Australia; 2 Office of the Chief Health Officer, NSW Ministry of Health, Sydney, Australia; 3 Concord Repatriation General Hospital, Sydney, Australia; 4 Illawarra Local Aboriginal Lands Council, Wollongong, Australia; 5 Centre for Population Health, NSW Ministry of Health, Sydney, Australia; 6 Epidemiology Section, Population Health Protection and Prevention, ACT Health, Canberra, Australia; 7 Clinical Services, NSW Ambulance, Sydney, Australia; 8 ACT Chief Health Officer & Deputy Director-General, Population Health Protection and Prevention, ACT Health, Canberra, Australia; 9 Chief Health Officer, NSW Ministry of Health, Sydney, Australia

**Keywords:** ST-segment elevation myocardial infarction (STEMI), Percutaneous coronary intervention, Angiography

## Abstract

**Background:**

Timely restoration of bloodflow acute ST-segment elevation myocardial infarction (STEMI) reduces myocardial damage and improves prognosis. The objective of this study was describe the association of demographic factors with hospitalisation rates for STEMI and time to angiography, Percutaneous Coronary Intervention (PCI) and Coronary Artery Bypass Graft (CABG) in New South Wales (NSW) and the Australian Capital Territory (ACT), Australia.

**Methods:**

This was an observational cohort study using linked population health data. We used linked records of NSW and the ACT hospitalisations and the Australian Government Medicare Benefits Schedule (MBS) for persons aged 35 and over hospitalised with STEMI in the period 1 July 2010 to 30 June 2014. Survival analysis was used to determine the time between STEMI admission and angiography, PCI and CABG, with a competing risk of death without cardiac procedure.

**Results:**

Of 13,117 STEMI hospitalisations, 71% were among males; 55% were 65-plus years; 64% lived in major cities, and 2.6% were Aboriginal people. STEMI hospitalisation occurred at a younger age in males than females. Angiography and PCI rates decreased with age: angiography 69% vs 42% and PCI 60% vs 34% on day 0 for ages 35-44 and 75-plus respectively. Lower angiography and PCI rates and higher CABG rates were observed outside major cities. Aboriginal people with STEMI were younger and more likely to live outside a major city. Angiography, PCI and CABG rates were similar for Aboriginal and non-Aboriginal people of the same age and remoteness area.

**Conclusions:**

There is a need to improve access to definitive revascularisation for STEMI among appropriately selected older patients and in regional areas. Aboriginal people with STEMI, as a population, are disproportionately affected by access to definitive revascularisation outside major cities. Improving access to timely definitive revascularisation in regional areas may assist in closing the gap in cardiovascular outcomes between Aboriginal and non-Aboriginal people.

## Background

Ischaemic heart disease is the leading cause of death in Australia for both men and women [1]. For patients experiencing acute myocardial infarction (AMI), timely restoration of blood flow and subsequent revascularisation if required is important to minimise damage to the heart and improve prognosis. Revascularisation can be achieved by fibrinolytic therapy, percutaneous coronary intervention (PCI) or coronary artery bypass graft (CABG). There are well-established guidelines for management of Acute Coronary Syndrome (ACS) [2–5].

There are challenges for the health care system in making high quality care for AMI accessible to the whole population. For people living outside major cities, accessing coronary procedures within recommended times is challenging. High rates of comorbidities among ACS patients impact on the extent to which clinical management guidelines can be universally applied [6]. Lower rates of cardiac revascularisation procedures have been reported among Aboriginal compared to non-Aboriginal people [7]. Socio-economic status and private health insurance also influence access to coronary procedures [8].

ST segment elevation myocardial infarction (STEMI) is the most severe form of AMI, with higher risks of complications and early mortality compared to non-STEMI. The aim of this study was to examine the association of demographic factors with time (days) to angiography, and definitive revascularisation with PCI and CABG, for people hospitalised for STEMI in NSW and the ACT.

## Methods

### Study design

Observational cohort study using linked population health data.

### Setting

Australia has a universal health care system with free public acute hospital services and a large private sector including private hospitals and private care within the public hospitals. In 2011, NSW was home to one third of the Australian population (7.8 million people), including 4.2 million (54%) aged over 35, 5.8 million (75%) living in a major city, and more than 200,000 (2.9%) Aboriginal people, of whom 95,000 (45%) were living in a major city [9–11]. The Australian Capital Territory (ACT) is a self-governing territory of Australia enclaved within NSW and in 2011, was home to around 390,000 people [9]. The only city in the ACT is Canberra, the capital city of Australia. ACT Health provides services to people living the ACT and surrounding regions.

### Study population

People aged 35 years and over hospitalised with STEMI in NSW or ACT in the period 2010–11 to 2013–14.

### Data sources

De-identified linked records of the following data collections: NSW Admitted Patient Data Collection, ACT Admitted Patient Collection and the Medicare Benefits Schedule (MBS). For NSW hospitals, data for all public and private hospitals were included; for ACT, data for Canberra Hospital were included. For population information we used Australian Bureau of Statistics (ABS) population estimates and projections based on the 2011 Census [9, 11].

### Data linkage

Linkage of the NSW and ACT data collections was carried out by the Centre for Health Record Linkage [12]. Linkage of the MBS data was carried out by the AIHW Data Linkage Unit [13].

### Definitions

**STEMI hospitalisation:** a continuous period of hospital care, represented by linked contiguous hospital and MBS records that start on a hospital admission for acute care with a primary diagnosis of STEMI (ICD-10-AM code I21.0 – I21.3).

**Cardiac procedures:** procedure codes are shown in Additional file 1. Where a PCI was recorded, angiography was considered to have been carried out.

**Time to cardiac procedure:** the elapsed days between the date of initial hospital admission for STEMI and the date of cardiac procedure. Where the procedure date was not available (11% of angiographies, 24% of PCIs and 5% of CABGs), the time to procedure was derived from the median time to procedure for individual hospital records where procedure dates were known by age/remoteness/Aboriginality group. The estimated median was 0 days for 98.3% of angiographies and 98.0% of PCIs. Rates of angiography/PCI on the day of presentation (day 0) and angiography/PCI/CABG by the end of day 7 following hospital admission are presented.

**Aboriginal** includes both Aboriginal and Torres Strait Islander people. An Enhanced Reporting of Aboriginality (ERA) variable was created using a weight of evidence from linked records for each person to correct for the under-reporting of Aboriginal people on administrative health data [14].

**Geographic Remoteness** ARIA category of the Statistical Local Area of residence in 2011 [15].

### Statistical analysis

Population-based age-specific rates of STEMI hospitalisation were calculated using ages grouped in 10-year intervals to 75-plus. Survival analysis, with a competing risk of death without cardiac procedure, was used to determine the time between STEMI admission and cardiac procedure; differences with a *p*-value greater than or equal to 0.01 are reported as different. Descriptive analyses were carried out in SAS 9.3 [16]. Survival analyses and figures were produced using R [17–19].

### Results

**Fig. 1:**
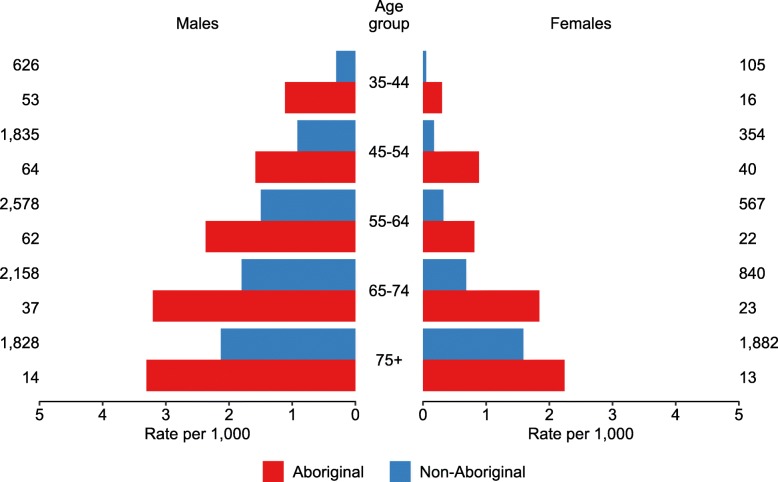
STEMI hospitalisations by Aboriginality, age and sex, NSW and ACT 2010–11 to 2013-14

In the four-year period 2010–11 to 2013–14 there were 13,117 STEMI hospitalisations in NSW and the ACT among people aged 35 years and over, of which: 71% were male; 52% were aged 65 years and over; 64% lived in major cities, 23% in inner regional areas and 7% in outer regional areas; and 344 (3%) were among Aboriginal people (Table 1). Age-specific population rates of STEMI hospitalisation were higher among Aboriginal people at all ages (Fig. 1). Aboriginal people with a STEMI hospitalisation were younger than non-Aboriginal people (75% under 65 versus 47%) and more likely to live outside a major city (65% versus 25%).

**Table 1:** Characteristics of STEMI hospitalisations

	Total	Aboriginal	Non-Aboriginal	*p*-value
Number	13,117	344	12,773	
Sex				
Male, N (%)	9,255 (70.6)	230 (66.9)	9,025 (70.7)	<0.001
Female, N (%)	3,862 (29.4)	114 (33.1)	3,748 (29.3)	
Age				
35-44, N (%)	800 (6.1)	69 (20.1)	731 (5.7)	<0.001
45-54, N (%)	2,293 (17.5)	104 (30.2)	2,189 (17.1)	
55-64, N (%)	3,229 (24.6)	84 (24.4)	3,145 (24.6)	
65-74, N (%)	3,058 (23.3)	60 (17.4)	2,998 (23.5)	
75+, N (%)	3,737 (28.5)	27 (7.8)	3,710 (29.0)	
Remoteness				
Major City, N (%)	8,735 (63.8)	115 (33.4)	8,620 (67.5)	<0.001
Inner Regional, N (%)	3,068 (23.4)	133 (38.7)	2,935 (23.0)	
Outer and remote, N (%)	924 (7.0)	89 (25.9)	835 (6.5)	

Overall, of 13,117 STEMI hospitalisations, 7,563 (58%) received angiography and 6,446 (49%) received PCI on the day of admission to hospital (day 0). By the end of the seventh day following admission (day 7) 10,766 (82%) received angiography, 8,483 (65%) received PCI and 546 (4%) received a CABG.

#### Time to angiography

**Fig. 2:**
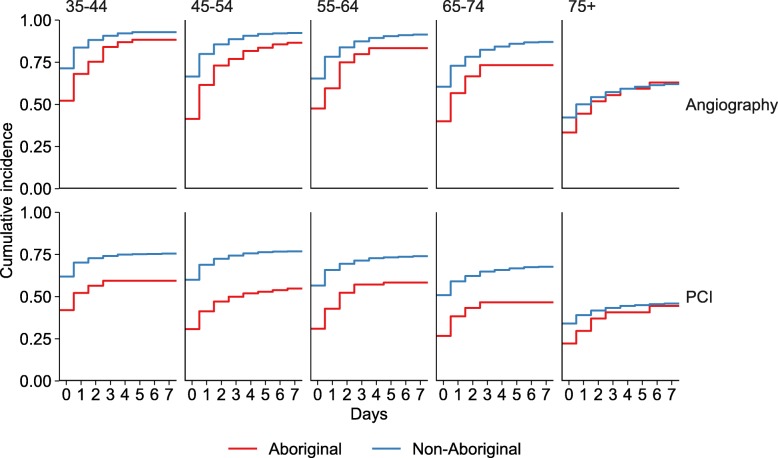
Time to cardiac procedure following STEMI admission by Aboriginality and age, NSW and ACT 2010–11 to 2013–14

Angiography rates decreased with age. For people aged 35–44, 70% received angiography on day 0 (day 7: 92%), compared to 42% on day 0 (day 7: 61%) for those aged 75-plus. Lower angiography rates were found among Aboriginal people than non-Aboriginal people at all ages except for patients over 75 years in whom rates were similar (Fig. 2, Additional Table A2-2).

**Fig. 3:**
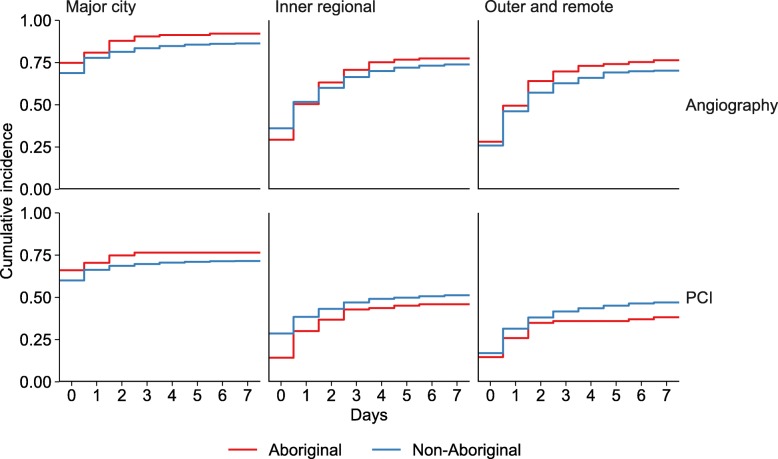
Time to cardiac procedure following STEMI admission by Aboriginality and geographic remoteness, NSW and ACT 2010–11 to 2013–14

Lower angiography rates were found in those living outside major cities compared with major cities: for residents of major cities, 69% received angiography on day 0 (day 7: 86%), compared with 36% (day 7: 74%) for inner regional areas and 26% (day 7: 71%) for more remote areas (Fig. 3, Additional Table A2-2).

**Fig. 4:**
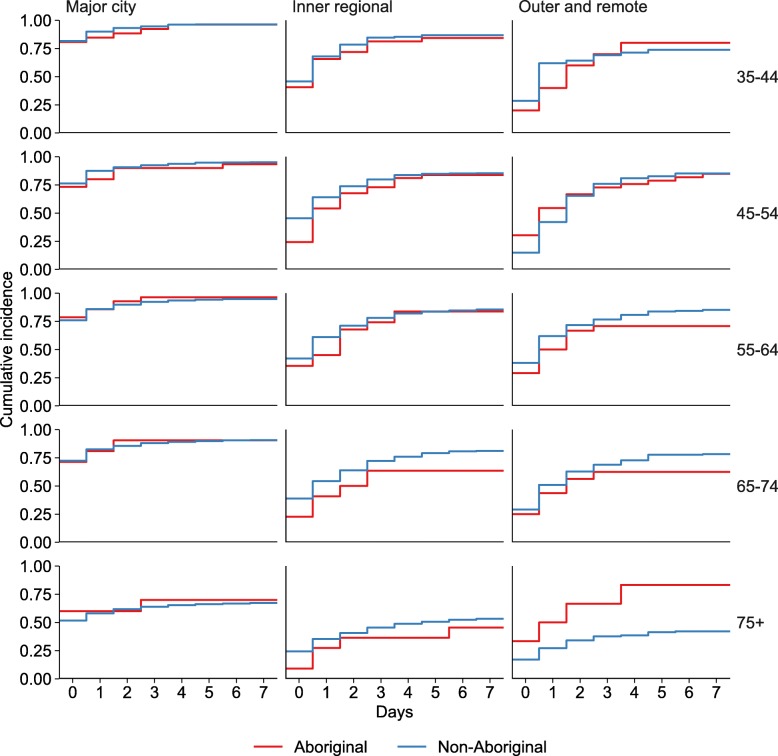
Time to Angiography following STEMI by Aboriginal, age and remoteness, NSW and ACT 2010–11 to 2013–14

When age and geographic remoteness were examined simultaneously, the highest angiography rate was in 35–44 year olds living in major cities (day 0: 82%, day 7: 96%). There was no difference in rates of angiography between Aboriginal and non-Aboriginal people of the same age and living in the same remoteness area (Fig. 4, Additional Table A2-2).

#### Time to PCI

PCI rates decreased with age. PCI rates on day 0 ranged from 60% in people aged 35–44 years to 34% for those aged 75-plus (Fig. 2, Additional Table A2-3). By the end of day 7 the PCI rate in people aged 35–44 was 74% compared with 46% among those aged 75-plus. Among people under 75 years, Aboriginal people received fewer PCIs than non-Aboriginal people of the same age.

People living outside major cities received fewer PCIs compared with residents of major cities: in major cities, 60% received PCI on day 0 (day 7: 72%), compared with 28% (day 7: 51%) for inner regional areas and 17% (day 7: 46%) for more remote areas. Higher PCI rates were found among Aboriginal people than non-Aboriginal in major cities, but were similar for Aboriginal and non-Aboriginal people living more remotely (Fig. 3, Additional Table A2-3).

**Fig. 5:**
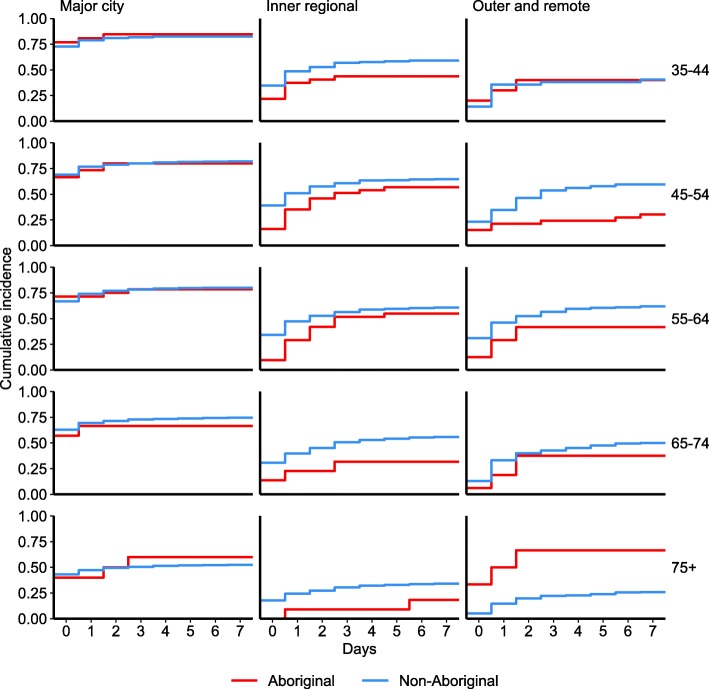
Time to PCI following STEMI by Aboriginal, age and remoteness, NSW and ACT 2010–11 to 2013–14

There was no difference in PCI rates between Aboriginal and non-Aboriginal people of the same age and living in the same remoteness area (Fig. 5, Additional Table A2-3).

#### Time to CABG

CABG rates for people under 75 years increased with age. CABG rates by the end of day 7 of STEMI hospitalisation ranged from 3% in those aged 35–44 years to 6% in 64–75 year olds.

CABG rates by the end of day seven were similar regardless of where people lived: 4% for major cities and inner regional areas, and 5% for more remote areas. In outer regional and remote areas, the CABG rate was slightly higher among Aboriginal people (9%) than non-Aboriginal people (5%).

CABG rates were similar for Aboriginal people and non-Aboriginal people of the same age and living in the same remoteness area.

## Discussion

This study of 13,117 STEMI hospitalisations in NSW/ACT found that angiography and PCI rates were higher in younger people, residents of major cities, and non-Aboriginal people, while CABG rates showed the opposite pattern. There was no difference in angiography and PCI rates between Aboriginal and non-Aboriginal people of the same age and living in the same level of geographic remoteness. Age-specific population rates of STEMI hospitalisation were higher among Aboriginal people at all ages.

Public hospitals rely on a networked referral system to deliver cardiac procedures following AMI. The inclusion of linked data for both NSW and the ACT enabled us to examine access to cardiac procedures at a population level and gave a more complete picture of cardiac procedures than has previously been available. While non-admitted patient records for NSW and ACT were not available for this study, linked MBS data contributed information on cardiac procedures carried out in the non-admitted setting. The lower PCI and higher CABG rates for older people that we observed are probably related to more severe coronary artery disease at older ages and presence of comorbidities [6]. A lower PCI rate for AMI among Aboriginal than non-Aboriginal people was reported in NSW in 2000–2008 [8]. This discrepancy persisted after adjusting for various factors including geographic remoteness, a finding that was not confirmed in our study. This is probably due to better enumeration of cardiac procedures with the inclusion of ACT and MBS data in our study, and improved availability of cardiac catheter laboratories in NSW regional areas during our later study period. Other population-based studies have not found a relationship between geographic remoteness and age-standardised rates of coronary angiography across Australia [20]; however, the use of unlinked data in these studies is likely to have affected results.

The higher rate of STEMI hospitalisations among Aboriginal people at all ages is related to higher rates of risk factors for ischaemic heart disease, including smoking, insufficient physical activity, overweight and obesity, diabetes and high blood pressure [21]. In 2013, the Australian Health Ministers’ Advisory Council established the Better Cardiac Care for Aboriginal and Torres Strait Islander People Project. Improving access to timely definitive revascularisation procedures is a key priority [22, 23]. For people of the same age living in the same geographic area, we found similar rates of cardiac revascularisation procedures for Aboriginal and non-Aboriginal people. At the 2011 Census, over half (53%) Aboriginal people aged over 35 in NSW and ACT lived outside major cities compared with around a quarter (26%) of non-Aboriginal people [11]; similarly Aboriginal people hospitalised for STEMI were more likely to live outside major cities (65%) than non-Aboriginal people (25%). In addition to having higher rates of risk factors for ischaemic heart disease, Aboriginal people as a population are disproportionately affected by difficulties with access to definitive revascularisation outside major cities.

There is room for improvement in access to definitive revascularisation of patients with STEMI living in regional areas. The National Heart Foundation [24] provides an easy-to-follow guide on when to call an ambulance for heart attack. The NSW State Reperfusion Strategy includes: pre-hospital assessment for primary angioplasty, whereby a patient with confirmed STEMI patient is immediately transported to a cardiac catheterisation laboratory, bypassing other hospitals; pre-hospital thrombolysis administered by paramedics; and clinical support and nurse administered thrombolysis for small hospitals [25]. Pre-hospital thrombolysis has been demonstrated to be safe and effective in circumstances where transport distance to a cardiac catheterisation laboratory are great [26]. However, even where thrombolysis is administered, the patient still requires transport to a cardiac catheterisation laboratory for investigation and definitive care.

The limitations of the study include:

- The true rate of procedures for residents of regional areas is probably slightly higher than we found due to other interstate patient flows for cardiac procedures.
- For STEMI, emergency reperfusion therapy with either PCI or fibrinolytic therapy is recommended [27]. Information on fibrinolysis was not available for this study.
- The linked dataset contained information on dates but not times, so we could not assess time to cardiac procedures in hours.
- Aboriginal people are under-reported on administrative data collections. It is estimated that 75% of Aboriginal people were correctly reported as Aboriginal on NSW Admitted Patient data in 2012–13 [28]. By comparison, the Australian Census found that the NSW Aboriginal population increased by 25% between the 2006 and 2011 Censuses, and again between the 2011 and 2016 Censuses, with almost half of this probably associated with an increased propensity for Aboriginal people to report themselves as Aborignal [29, 30]. While we have corrected for under-reporting on the linked health dataset using ERA, it is possible that some under-reporting remains and the disparities in STEMI rates between Aboriginal and non-Aboriginal people are actually greater than we found.
- Administrative data collections do not contain clinical information, such as troponin levels, which help determine the treatment pathway [31].

A national approach to data linkage would enable all states and territories to obtain an accurate view of the quality of care for STEMI for their populations, including cross-border flows. Inclusion of ambulance data in future linkages would enable assessment of pre-hospital access to thrombolysis, pre-hospital assessment for primary angioplasty, and time to angiography. The increasing use of electronic medical records may provide a mechanism for a sample of case records to be included in record linkage studies, enabling relevant clinical information on acute care to supplement the breadth of information currently available in linked health administrative records.

## Conclusion

There is room for improvement in access to definitive revascularisation for STEMI for appropriately selected older patients and those living in regional areas. The use of cross-jurisdictional linked data, including MBS data, provides a population-based view and a more complete and accurate picture of cardiac procedures following STEMI than has previously been available.

## Supplementary information

**Additional file 1** Procedure-codes.pdf.

**Additional file 2** Additional-results-revised.pdf.

## Data Availability

The datasets generated during the current study are not publicly available as they contain information that could potentially re-identify individuals, but are available from the corresponding author and with relevant ethical approval on reasonable request.

## Abbreviations

ABS: Australian Bureau of Statistics
ACS: Acute Coronary Syndrome
ACT: Australian Capital Territory
AMI: Acute Myocardial Infarction
ERA: Enhanced Reporting of Aboriginality
CABG: Coronary Artery Bypass Graft
MBS: Medicare Benefits Schedule
NSW: New South Wales
PCI: Percutaneous Coronary Intervention
STEMI: ST-Elevation Myocardial Infarction
