# Supplementary material for: Cardiac procedures in ST-segment-elevation myocardial infarction - the influence of age, geography and Aboriginality
Source: BMC Cardiovasc Disord. 2020 May 14;20:224. doi: 10.1186/s12872-020-01487-0 (PMC7227061; doi:10.1186/s12872-020-01487-0)
Supplement: Supplementary file 1 — Additional file 1 Procedure-codes.pdf. [file 12872_2020_1487_MOESM1_ESM.pdf]

Appendix 1: Cardiac Procedure codes

NSW and ACT hospital data

Angiography

38215-00	38218-00	38218-01	38218-02

PCI

38300-00	38303-00	38306-00	38306-01	38306-02	38309-00	38312-00
38312-01	38315-00	38318-00	38318-01	90218-00	90218-01	90218-02
90218-03	90218-01					

CABG

38300-01	38303-01	38306-03	38306-04	38306-05	38497-00	38497-01
38497-02	38497-03	38497-04	38497-05	38497-06	38497-07	38500-00
38500-01	38500-02	38500-03	38500-04	38500-05	38503-00	38503-01
38503-02	38503-03	38503-04	38503-05	38505-00	90201-00	90201-01
90201-02	90201-03					

Medicare Benefits Schedule

Angiography

**Category**	3 Therapeutic Procedures
**Group**	T8 Surgical Operation
**Subgroup**	6. Cardio-thoracic
**Subheading**	Cardiology procedures
**Codes**	38215 – 38246

PCI

**Category**	3 Therapeutic Procedures
**Group**	T8 Surgical Operation
**Subgroup**	6. Cardio-thoracic
**Subheading**	Endovascular interventional procedures
**Codes**	38300 – 38318

CABG

**Category**	3 Therapeutic Procedures
**Group**	T8 Surgical Operation
**Subgroup**	6. Cardio-thoracic
**Subheading**	Surgery for ischaemic heart disease
**Codes**	38497 – 38504
