# Supplementary material for: Cardiac procedures in ST-segment-elevation myocardial infarction - the influence of age, geography and Aboriginality
Source: BMC Cardiovasc Disord. 2020 May 14;20:224. doi: 10.1186/s12872-020-01487-0 (PMC7227061; doi:10.1186/s12872-020-01487-0)
Supplement: Supplementary file 2 — Additional file 2 Additional-results-revised.pdf. [file 12872_2020_1487_MOESM2_ESM.pdf]

Appendix 2: Supplementary tables

Table A2-1: STEMI hospitalisations by Aboriginality, age and remoteness, NSW and ACT 2010-11 to 2013-14

Age	Remoteness	Aboriginal No.	Non-Aboriginal No.	Total No.
35-44	Major city	26	519	545
	Inner regional	32	144	176
	Outer and remote	10	42	52
	All	69	731	800
45-54	Major city	30	1,584	1,614
	Inner regional	37	418	455
	Outer and remote	33	121	154
	All	104	2,189	2,293
55-64	Major city	28	2,141	2,169
	Inner regional	31	669	700
	Outer and remote	24	223	247
	All	84	3,145	3,229
65-74	Major city	21	1,945	1,966
	Inner regional	22	733	755
	Outer and remote	16	202	218
	All	60	2,998	3,058
75+	Major city	10	2,431	2,441
	Inner regional	11	971	982
	Outer and remote	6	247	253
	All	27	3,710	3,737
Total	Major city	115	8,620	8,735
	Inner regional	133	2,935	3,068
	Outer and remote	89	835	924
	All	344	12,773	13,117

Table A2-2: Time to Angiography following STEMI by Aboriginal, age and remoteness, NSW and ACT 2010-11 to 2013-14.

Age	Remote	Time days	Aboriginal No. (%)	Non-Aboriginal No. (%)	Total No. (%)	Comparison RR (95% CI)
35-44	Major city	0	21 (80.8)	424 (81.7)	445 (81.7)	1.01 (0.83, 1.23)
		7	25 (96.2)	500 (96.3)	525 (96.3)	1.00 (0.93, 1.08)
	Inner regional	0	13 (40.6)	66 (45.8)	79 (44.9)	1.13 (0.72, 1.78)
		7	27 (84.4)	125 (86.8)	152 (86.4)	1.03 (0.87, 1.21)
	Outer and remote	0	np (–)	np (–)	14 (26.9)	np (–)
		7	8 (80.0)	31 (73.8)	39 (75.0)	0.92 (0.64, 1.32)
	All	0	36 (52.2)	522 (71.4)	558 (69.8)	1.37 (1.09, 1.72)
		7	61 (88.4)	679 (92.9)	740 (92.5)	1.05 (0.96, 1.15)
45-54	Major city	0	22 (73.3)	1,209 (76.3)	1,231 (76.3)	1.04 (0.84, 1.29)
		7	28 (93.3)	1,507 (95.1)	1,535 (95.1)	1.02 (0.93, 1.12)
	Inner regional	0	9 (24.3)	190 (45.5)	199 (43.7)	1.87 (1.05, 3.33)
		7	31 (83.8)	357 (85.4)	388 (85.3)	1.02 (0.88, 1.18)
	Outer and remote	0	10 (30.3)	18 (14.9)	28 (18.2)	0.49 (0.25, 0.96)
		7	28 (84.8)	103 (85.1)	131 (85.1)	1.00 (0.85, 1.18)
	All	0	43 (41.3)	1,456 (66.5)	1,499 (65.4)	1.61 (1.28, 2.03)
		7	90 (86.5)	2,021 (92.3)	2,111 (92.1)	1.07 (0.99, 1.15)
55-64	Major city	0	22 (78.6)	1,627 (76.0)	1,649 (76.0)	0.97 (0.80, 1.18)
		7	27 (96.4)	2,031 (94.9)	2,058 (94.9)	0.98 (0.92, 1.06)
	Inner regional	0	11 (35.5)	281 (42.0)	292 (41.7)	1.18 (0.73, 1.92)
		7	26 (83.9)	572 (85.5)	598 (85.4)	1.02 (0.87, 1.19)
	Outer and remote	0	7 (29.2)	85 (38.1)	92 (37.2)	1.31 (0.69, 2.49)
		7	17 (70.8)	190 (85.2)	207 (83.8)	1.20 (0.93, 1.56)
	All	0	40 (47.6)	2,055 (65.3)	2,095 (64.9)	1.37 (1.09, 1.72)
		7	70 (83.3)	2,875 (91.4)	2,945 (91.2)	1.10 (1.00, 1.21)
65-74	Major city	0	15 (71.4)	1,409 (72.4)	1,424 (72.4)	1.01 (0.77, 1.33)
		7	19 (90.5)	1,764 (90.7)	1,783 (90.7)	1.00 (0.87, 1.15)
	Inner regional	0	np (–)	np (–)	290 (38.4)	np (–)
		7	14 (63.6)	595 (81.2)	609 (80.7)	1.28 (0.93, 1.75)
	Outer and remote	0	np (–)	np (–)	63 (28.9)	np (–)
		7	10 (62.5)	158 (78.2)	168 (77.1)	1.25 (0.85, 1.84)
	All	0	24 (40.0)	1,813 (60.5)	1,837 (60.1)	1.51 (1.11, 2.06)
		7	44 (73.3)	2,609 (87.0)	2,653 (86.8)	1.19 (1.02, 1.38)
75+	Major city	0	6 (60.0)	1,257 (51.7)	1,263 (51.7)	0.86 (0.52, 1.43)
		7	7 (70.0)	1,634 (67.2)	1,641 (67.2)	0.96 (0.64, 1.44)
	Inner regional	0	np (–)	np (–)	238 (24.2)	np (–)
		7	5 (45.5)	518 (53.3)	523 (53.3)	1.17 (0.61, 2.25)
	Outer and remote	0	np (–)	np (–)	44 (17.4)	np (–)
		7	np (–)	np (–)	109 (43.1)	np (–)
	All	0	9 (33.3)	1,565 (42.2)	1,574 (42.1)	1.27 (0.74, 2.16)
		7	17 (63.0)	2,300 (62.0)	2,317 (62.0)	0.98 (0.74, 1.32)
Total	Major city	0	86 (74.8)	5,926 (68.7)	6,012 (68.8)	0.92 (0.83, 1.02)
		7	106 (92.2)	7,436 (86.3)	7,542 (86.3)	0.94 (0.89, 0.99)
	Inner regional	0	39 (29.3)	1,059 (36.1)	1,098 (35.8)	1.23 (0.94, 1.61)
		7	103 (77.4)	2,167 (73.8)	2,270 (74.0)	0.95 (0.87, 1.05)
	Outer and remote	0	25 (28.1)	216 (25.9)	241 (26.1)	0.92 (0.65, 1.31)
		7	68 (76.4)	586 (70.2)	654 (70.8)	0.92 (0.81, 1.04)
	All	0	152 (44.2)	7,411 (58.0)	7,563 (57.7)	1.31 (1.16, 1.48)
		7	282 (82.0)	10,484 (82.1)	10,766 (82.1)	1.00 (0.95, 1.05)

Table A2-3: Time to PCI following STEMI by Aboriginal, age and remoteness, NSW and ACT 2010-11 to 2013-14.

Age	Remote	Time days	Aboriginal No. (%)	Non-Aboriginal No. (%)	Total No. (%)	Comparison RR (95% CI)
35-44	Major city	0	20 (76.9)	378 (72.8)	398 (73.0)	0.95 (0.76, 1.18)
		7	22 (84.6)	428 (82.5)	450 (82.6)	0.97 (0.82, 1.15)
	Inner regional	0	7 (21.9)	50 (34.7)	57 (32.4)	1.59 (0.79, 3.17)
		7	14 (43.8)	85 (59.0)	99 (56.3)	1.35 (0.89, 2.04)
	Outer and remote	0	np (–)	np (–)	8 (15.4)	np (–)
		7	np (–)	np (–)	21 (40.4)	np (–)
	All	0	29 (42.0)	453 (62.0)	482 (60.2)	1.47 (1.11, 1.96)
		7	41 (59.4)	552 (75.5)	593 (74.1)	1.27 (1.04, 1.55)
45-54	Major city	0	20 (66.7)	1,093 (69.0)	1,113 (69.0)	1.04 (0.80, 1.34)
		7	24 (80.0)	1,297 (81.9)	1,321 (81.8)	1.02 (0.85, 1.23)
	Inner regional	0	6 (16.2)	163 (39.0)	169 (37.1)	2.40 (1.14, 5.05)
		7	21 (56.8)	270 (64.6)	291 (64.0)	1.14 (0.85, 1.52)
	Outer and remote	0	5 (15.2)	28 (23.1)	33 (21.4)	1.53 (0.64, 3.65)
		7	10 (30.3)	72 (59.5)	82 (53.2)	1.96 (1.15, 3.36)
	All	0	32 (30.8)	1,315 (60.1)	1,347 (58.7)	1.95 (1.46, 2.61)
		7	57 (54.8)	1,683 (76.9)	1,740 (75.9)	1.40 (1.18, 1.67)
55-64	Major city	0	20 (71.4)	1,432 (66.9)	1,452 (66.9)	0.94 (0.74, 1.19)
		7	22 (78.6)	1,715 (80.1)	1,737 (80.1)	1.02 (0.84, 1.24)
	Inner regional	0	np (–)	np (–)	231 (33.0)	np (–)
		7	17 (54.8)	407 (60.8)	424 (60.6)	1.11 (0.80, 1.54)
	Outer and remote	0	np (–)	np (–)	72 (29.1)	np (–)
		7	10 (41.7)	138 (61.9)	148 (59.9)	1.49 (0.91, 2.41)
	All	0	26 (31.0)	1,781 (56.6)	1,807 (56.0)	1.83 (1.33, 2.52)
		7	49 (58.3)	2,327 (74.0)	2,376 (73.6)	1.27 (1.06, 1.52)
65-74	Major city	0	12 (57.1)	1,223 (62.9)	1,235 (62.8)	1.10 (0.76, 1.60)
		7	14 (66.7)	1,451 (74.6)	1,465 (74.5)	1.12 (0.83, 1.52)
	Inner regional	0	np (–)	np (–)	229 (30.3)	np (–)
		7	7 (31.8)	410 (55.9)	417 (55.2)	1.76 (0.95, 3.25)
	Outer and remote	0	np (–)	np (–)	27 (12.4)	np (–)
		7	6 (37.5)	101 (50.0)	107 (49.1)	1.33 (0.70, 2.55)
	All	0	16 (26.7)	1,525 (50.9)	1,541 (50.4)	1.91 (1.25, 2.91)
		7	28 (46.7)	2,031 (67.7)	2,059 (67.3)	1.45 (1.11, 1.90)
75+	Major city	0	np (–)	np (–)	1,056 (43.3)	np (–)
		7	6 (60.0)	1,275 (52.4)	1,281 (52.5)	0.87 (0.53, 1.45)
	Inner regional	0	np (–)	np (–)	174 (17.7)	np (–)
		7	np (–)	np (–)	333 (33.9)	np (–)
	Outer and remote	0	np (–)	np (–)	15 (5.9)	np (–)
		7	np (–)	np (–)	68 (26.9)	np (–)
	All	0	6 (22.2)	1,263 (34.0)	1,269 (34.0)	1.53 (0.76, 3.11)
		7	12 (44.4)	1,703 (45.9)	1,715 (45.9)	1.03 (0.68, 1.58)
Total	Major city	0	76 (66.1)	5,178 (60.1)	5,254 (60.1)	0.91 (0.80, 1.04)
		7	88 (76.5)	6,166 (71.5)	6,254 (71.6)	0.93 (0.84, 1.04)
	Inner regional	0	19 (14.3)	841 (28.7)	860 (28.0)	2.01 (1.32, 3.05)
		7	61 (45.9)	1,503 (51.2)	1,564 (51.0)	1.12 (0.93, 1.35)
	Outer and remote	0	13 (14.6)	142 (17.0)	155 (16.8)	1.16 (0.69, 1.97)
		7	34 (38.2)	392 (46.9)	426 (46.1)	1.23 (0.93, 1.62)
	All	0	109 (31.7)	6,337 (49.6)	6,446 (49.1)	1.57 (1.34, 1.83)
		7	187 (54.4)	8,296 (64.9)	8,483 (64.7)	1.19 (1.08, 1.32)
